# Impact of Plasma Surface Treatment on Implant Stability and Early Osseointegration: A Retrospective Cohort Study

**DOI:** 10.3390/ma18194568

**Published:** 2025-09-30

**Authors:** Yoon-Kyung Kim, Hyunsuk Choi, Hyung-Gyun Kim, Dong-Seok Sohn

**Affiliations:** 1 Department of Dentistry and Advanced General Dentistry, Daegu Catholic University Medical Center, Daegu 42472, Republic of Korea; yooocc@gmail.com; 2 Department of Dentistry and Prosthodontics, Daegu Catholic University School of Medicine, Daegu 42472, Republic of Korea; hschoi@cu.ac.kr; 3 Department of Dentistry and Advanced General Dentistry, Daegu Catholic University School of Medicine, Daegu 42472, Republic of Korea; hgkim25@cu.ac.kr; 4 Department of Dentistry and Oral and Maxillofacial Surgery, Daegu Catholic University School of Medicine, Daegu 42472, Republic of Korea

**Keywords:** plasma surface treatment, osseointegration, dental implant, hydrophilicity, resonance frequency analysis, implant stability quotient (ISQ), early loading

## Abstract

(1) Introduction: The clinical success of dental implants depends on rapid osseointegration, which can be impaired by hydrocarbon contamination and biological aging of titanium surfaces. Chairside plasma surface treatment has emerged as a practical method to restore surface hydrophilicity and enhance early bone–implant integration. (2) Materials and Methods: This retrospective cohort study evaluated 73 plasma-treated implants placed in 47 patients from June 2023 to October 2024. Non-thermal atmospheric pressure plasma was applied immediately before placement using the ACTILINK™ Reborn system. Implant stability was assessed baseline, weekly for the first four weeks, and again at week 8 using resonance frequency analysis (ISQ). Subgroup analyses were conducted according to initial ISQ, jaw location, implant length/diameter, and final insertion torque. (3) Results: All implants healed uneventfully without a stability dip. Mean ISQ increased from 78.97 ± 5.52 at placement to 83.74 ± 4.36 at week 8 (*p* < 0.001). Implants with lower initial stability demonstrated the greatest relative gains, while those with very high initial stability showed minimal changes. Mandibular and shorter implants demonstrated higher stability gains compared to maxillary and longer fixtures. (4) Conclusions: Chairside plasma surface treatment was associated with progressive ISQ increases during the 8-week healing period. The greatest gains occurred in implants with lower initial stability, while very stable implants showed little change. Stability improvements were also greater in mandibular sites, shorter fixtures, and those with higher insertion torque. These findings are limited to short-term ISQ outcomes and require validation in prospective controlled trials with standardized protocols.

## 1. Introduction

The clinical success of implant surgery is fundamentally dependent on effective osseointegration, which is strongly influenced by the surface properties of titanium-based implants [1]. Consequently, numerous surface modification techniques—particularly those designed to enhance surface roughness—have been developed to accelerate the biological process of osseointegration and facilitate immediate or early functional loading. Roughened implant surfaces outperform smooth surfaces by increasing the available bone–implant contact area and promoting cellular adhesion, proliferation, and osteogenic differentiation [2]. Among the various approaches, including acid etching, grit blasting, anodization, and calcium phosphate coating, the sandblasted, large-grit, acid-etched (SLA) technique has emerged as the clinical gold standard over the past several decades [3].

Despite the well-documented success of SLA implants, their biological performance is compromised by “biological aging”, largely attributable to carbon contamination [4]. Upon exposure to ambient air, hydrocarbons rapidly absorb to titanium surfaces, resulting in inevitable surface contamination [5]. As a result, most commercially available dental implants are presumed to be heavily coated with carbonaceous molecules by the time they are clinically applied [6]. This accumulation of surface carbon significantly diminishes protein adsorption, cellular attachment, proliferation, and differentiation—key processes required for robust osseointegration [5]. Experimental evidence demonstrates that, compared with pristine implant surfaces, those aged for just four weeks exhibit markedly reduced fibronectin and albumin adsorption, decreased numbers of adherent osteogenic cells, and limited areas of newly formed bone [5,6,7,8].

**Figure 1:**
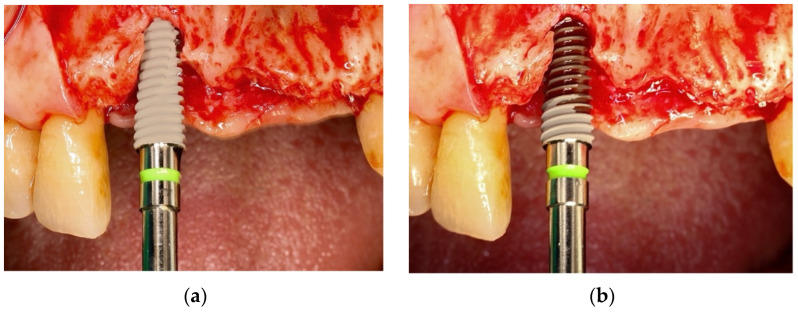
Representative example showing implant surface appearance before ((**a**), untreated) and after ((**b**), plasma-treated) chairside plasma activation. Plasma activation enhanced hemocompatibility, resulting in a blood-soaked appearance on the treated implant surface.

In addition to carbon contamination, the time-dependent accumulation of hydrocarbons on titanium implant surfaces induces a progressive loss of hydrophilicity, a property widely recognized as one of the most influential factors governing cell attachment [6,9]. Newly processed titanium surfaces typically display superhydrophilicity, with contact angles near 0°, but after only four weeks of ambient storage they undergo a marked transition toward hydrophobicity, with angles exceeding 60° [8]. Although the adoption of hydrophilicity as a direct indicator of bioactivity remains debated, it has been extensively investigated due to its close association with early cell–material interactions. Importantly, a clear inverse linear relationship between surface wettability and the number of adherent osteogenic cells has been demonstrated [6], and hydrophilic surfaces have also shown improved hemocompatibility [10] (Figure 1), which accelerates the osteogenic cascade by supporting early calcium and phosphate ion adsorption at the implant–blood interface [11]. Collectively, these findings suggest that maintaining or restoring hydrophilicity may be critical for optimizing early biological responses and achieving superior clinical outcomes in implant therapy.

As a result, surface decontamination or reactivation methods—most notably plasma treatment—have attracted increasing attention in recent years. Similar to ultraviolet photofunctionaliztion, plasma treatment is a post-manufacturing technique that can be applied chairside in clinical settings without altering implant topography [12,13]. Plasma, widely regarded as the fourth state of matter, is defined as an electrically charged gas created by applying high voltage or high temperature to specific gases such as O_2_, Ar, N_2_, and NH_3_, with the gas type determining the nature of reactive species incorporated onto the surface [12,14]. These reactive oxygen- or nitrogen-containing free radicals enhance surface decomposition capability, promote the removal of carbon contaminants, and increase wettability [15]. Mechanistically, reactive oxygen species generated during plasma exposure induce redox reactions that break carbon bonds in organic molecules, decompose contaminants through volatilization, and form hydrophilic hydroxyl groups, thereby reducing oxidative stress and the initial inflammatory response in peri-implant tissues [16,17]. Clinically, non-thermal (atmospheric pressure) plasma is particularly advantageous due to its portability, open-air applicability, and rapid activation time [12,18,19]. By restoring hydrophilicity without altering micro- or nanoscale roughness, plasma treatment strengthens the biological interface between titanium surfaces and surrounding bone and soft tissues, ultimately supporting improved osseointegration [13,20].

Numerous studies have investigated the effects of plasma treatment on titanium surfaces, particularly regarding the biological responses of surrounding tissues. In vitro, Ujino et al. demonstrated that atmospheric pressure plasma treatment increased bovine serum albumin (BSA) adsorption and enhanced rat bone marrow (RBM) cell adhesion on titanium disks [17]. Since albumin prevents the adsorption of pro-inflammatory and bacteria-associated proteins, its preferential adsorption plays a pivotal role in promoting favorable osseointegration [21]. Plasma-activated surfaces further exhibited denser attachment of osteoblasts and fibroblasts, elevated alkaline phosphatase (ALP) activity, and increased expression of transcription factors essential for osteoblastic differentiation [17,22]. In addition, systematic reviews have reported that cold atmospheric plasma exerts antimicrobial activity on titanium surfaces, suppressing oral pathogens such as *Streptococcus mitis* and *Staphylococcus aureus* and disrupting biofilms implicated in peri-implantitis [23]. At the in vivo level, Tsujita et al. reported consistent findings using plasma-treated titanium screws implanted in rat femurs, observing elongation of cell processes—an indicator of improved cell adhesion—together with reduced reactive oxygen species (ROS) levels and diminished carbon peaks, confirming effective surface decontamination [10]. Similarly, Kahm et al. demonstrated in a rabbit femur model that vacuum plasma treatment of SLA implants improved bone parameters and ISQ values, thereby further supporting the biological plausibility of plasma activation [24]. Collectively, these in vitro and in vivo findings are indicative of a favorable environment created by plasma treatment, with enhanced protein adsorption, osteogenic cell activity, and reduced oxidative stress supporting osseointegration.

Building upon this preclinical foundation, recent clinical investigations have also begun to validate the benefits of chairside plasma treatment in patients. For example, a prospective randomized controlled trial demonstrated that ACTILINK plasma activation enhanced osseointegration and bone regeneration in patients [18]. In addition, Canullo et al. conducted a randomized controlled trial with 5-year follow-up showing that argon plasma cleaning of implant abutments resulted in significantly less marginal bone loss compared with steam cleaning, thereby underscoring the clinical relevance of plasma activation in maintaining peri-implant tissue stability [25]. Furthermore, a systematic review confirmed that adjunctive plasma or UV activation can improve early implant stability compared with untreated controls [26]. Together, these findings highlight the growing body of contemporary clinical evidence supporting plasma surface activation as a practical adjunct to conventional implant therapy.

The aim of the present study was to determine whether the favorable biological effects of plasma surface activation, previously demonstrated in vitro and in vivo, are reproducible in clinical practice. Specifically, we sought to evaluate whether plasma treatment could stabilize implant stability during the early healing phase, thereby enabling functional loading as early as the fourth postoperative week. Early loading protocols require reliable primary stability, and clinical guidelines have emphasized that implant surface modifications can contribute to more predictable osseointegration and treatment success [27,28]. Although plasma treatment has recently gained attention, patient-based follow-up studies remain limited. In this investigation, plasma surface activation was performed using a novel device, the ACTILINK™ Reborn (Plasmapp Co., Ltd., Daejeon, Republic of Korea), which generates plasma under optimized vacuum conditions (5–10 Torr) to maximize hydrocarbon removal efficiency. This compact, chairside-compatible system (170 mm W × 266 mm D × 346 mm H) was specifically engineered for convenient clinical use [29]. In a prior animal study, application of the ACTILINK™ system resulted in approximately 58% reduction in hydrocarbon contamination, a 25% increase in protein adsorption, and a 39% enhancement in cell attachment, collectively accelerating osseointegration in rabbit models [30]. Building on this preclinical evidence, the present study aims to provide patient-based clinical data supporting the biological and practical benefits of chairside plasma treatment in dental implantology.

## 2. Materials and Methods

Ethics statement. This retrospective study was approved by the Institutional Review Board of Daegu Catholic University Medical Center (IRB No. 2025-06-021), and all procedures were performed in accordance with the ethical standards of the institutional and/or national research committee and with the 1964 Helsinki declaration and its later amendments. The requirement for informed consent was waived due to the retrospective nature of the study.

Patients and case selection. From June 2023 to October 2024, forty-seven patients who underwent implant surgery at the Department of Dentistry, Daegu Catholic University Medical Hospital, were included in this study. A total of 73 implants were placed, comprising 28 in the maxilla and 45 in the mandible (Table 1). Patients were enrolled irrespective of age or sex, except for those presenting systemic conditions known to critically impair osseointegration (e.g., uncontrolled diabetes, metabolic bone disease). Individuals unable to attend weekly follow-up visits due to physical limitations or geographic constraints were also excluded. Notably, smokers and bruxers were not excluded. This retrospective cohort study involved only implants that had undergone plasma surface treatment. To ensure the feasibility of early functional loading at the fourth postoperative week, implants were included only if they achieved a primary stability of ≥35 N·cm at placement, measured using a manual ratchet. A broad spectrum of cases was incorporated within these parameters, including those involving extensive bone augmentation (*n* = 27), sinus elevation (*n* = 7), and immediate placement in fresh extraction sockets (*n* = 18). Because of the retrospective nature of this study, variability in patient data collection and case standardization could not be fully controlled, which should be considered when interpreting the outcomes.

Implant systems. Four distinct implant systems were used in this study: Biotem Implant Fixture (Biotem Implant Co., Ltd., Hanam-si, Gyeonggi-do, Republic of Korea); OsseoSpeed™ (Astra Tech Implant System, Dentsply Sirona, Mölndal, Sweden); AnyOne Internal Fixture (Megagen Implant Co., Ltd., Daegu, Republic of Korea); and IS-II Active Fixture (Neo Biotech Co., Ltd., Seoul, Republic of Korea). The surface characteristics varied according to the manufacturer. Biotem implants featured a conventional sandblasted, large-grit, acid-etched (SLA) surface without further chemical modifications. Megagen implants employed the XPEED^®^ surface, based on the SLA protocol but enhanced with calcium ion incorporation to accelerate bone healing [31]. Neo Biotech’s IS-II Active implants also utilized a conventional SLA surface without additional surface agents. In contrast, Astra Tech’s OsseoSpeed™ implants were treated with a proprietary fluoride-modified titanium surface designed to promote early osseointegration.

Due to the retrospective design of this study, implant systems could not be standardized. Nevertheless, three of the four systems were based on SLA-type surface modification, which is widely accepted as a benchmark approach for enhancing osseointegration [3]. Although OsseoSpeed™ implants differ in utilizing a fluoride-modified surface produced through TiO_2_ sandblasting and hydrofluoric acid etching, their resulting surface roughness has been reported to fall within the range observed for SLA-treated implants [32]. Importantly, irrespective of the specific pre-packaging surface modifications, all titanium implant surfaces are inevitably exposed to ambient air before placement, leading to hydrocarbon accumulation that reduces surface biocompatibility [27].

Plasma activation cycle. Immediately before surgical placement, each dental implant underwent a 1 min plasma activation using the ACTILINK™ Reborn device (Plasmapp Co., Ltd., Daejeon, Republic of Korea). The system is equipped with three independent plasma modules, supported by a shared vacuum pump and pressure gauge, allowing plasma to be generated independently within each module [4]. A fixture driver holder located at the center of the device secures the implant fixture during treatment. Once the fixture is seated, a cylindrical Pyrex^®^ component descends to connect with a silicone stopper, sealing the chamber from ambient air and creating a partial vacuum [30].

Each activation cycle lasts 60 s and consists of four sequential phases: (1) vacuum formation (30 s), during which a base pressure of 5 Torr is established via the vacuum pump; (2) plasma exposure (8 s), generated by a powered electrode at the top of the chamber and applied directly to the implant surface; (3) decontamination (17 s), in which residual surface impurities are removed through the vacuum port; and (4) venting (5 s), which evacuates the gas from the chamber [30]. This process enables efficient removal of hydrocarbon contaminants while preserving the original surface topography of the implant.

Surgical procedures and prosthesis delivery. Routine local anesthesia was administered, and full-thickness mucoperiosteal flaps were elevated. Final drilling was performed using a drill 1.0 mm smaller than the intended implant diameter, and implants were placed with their coronal margin positioned 2 mm below the proximal bone crest. During drilling, each implant surface underwent immediate plasma activation using the ACTILINK™ Reborn device, with two consecutive treatment cycles applied to every fixture. In cases requiring bone grafting, Sticky Bone™—a combination of particulate graft material and autologous fibrin glue prepared from the patient’s blood—was utilized. The final insertion torque was measured with a calibrated torque wrench (LASAK Ltd., Prague, Czech Republic) in accordance with the manufacturer’s protocol. Postoperatively, patients received amoxicillin–clavulanic acid (500/125 mg) three times daily for infection control.

All sutures were removed two weeks after surgery. At three weeks postoperatively, impressions were taken for provisional restorations, which were delivered at week 4. Following one month of provisional prosthesis use, definitive impressions were obtained, and final prostheses were delivered at eight weeks postoperatively.

Implant stability measurements. To quantitatively evaluate implant stability, resonance frequency analysis (RFA) was performed at baseline (immediately after placement) and at postoperative weeks 1, 2, 3, 4, and up to week 8 using the MEGA ISQ™ device (Megagen Implant Co., Ltd., Daegu, Republic of Korea). A transducer peg (Mega ISQ Peg) was hand-tightened into each implant fixture using finger force, applying an estimated torque of approximately 4–6 N·cm, as recommended by the manufacturer. For implants restored with provisional prostheses, the prostheses were temporarily removed prior to measurement. During assessment, the probe was positioned 1–2 mm from the peg, oriented perpendicular to its longitudinal axis, and maintained without direct contact [33]. At each time point, three ISQ values were obtained—one each from the buccal, lingual, and mesial directions—and the mean value was recorded to two decimal places. All ISQ measurements were performed by the same experienced clinician throughout the study period, following a standardized protocol. Because this was a retrospective single-arm design without a comparator group, examiner blinding was not applicable; however, using a single operator minimized inter-operator variability and ensured consistency in measurement.

Statistical analysis. All statistical analyses were performed using IBM SPSS Statistics for Windows, version 29.0 (IBM Corp., Armonk, NY, USA). Time-dependent changes in implant stability were assessed using repeated-measures analysis of variance (ANOVA). When significant differences were observed, pairwise comparisons were conducted with paired *t*-tests, and Bonferroni correction was applied to adjust for type I error in post hoc analysis. Time-dependent ISQ changes were also visualized with line graphs. Results are expressed as mean ± standard deviation (SD), accompanied by 95% confidence intervals (95% CI) to indicate the precision of the estimates. Because this study was retrospective in nature, no a priori sample size calculation was performed. A *p*-value < 0.05 was considered statistically significant.

## 3. Results

### 3.1. Overall Tendency

**Figure 2:**
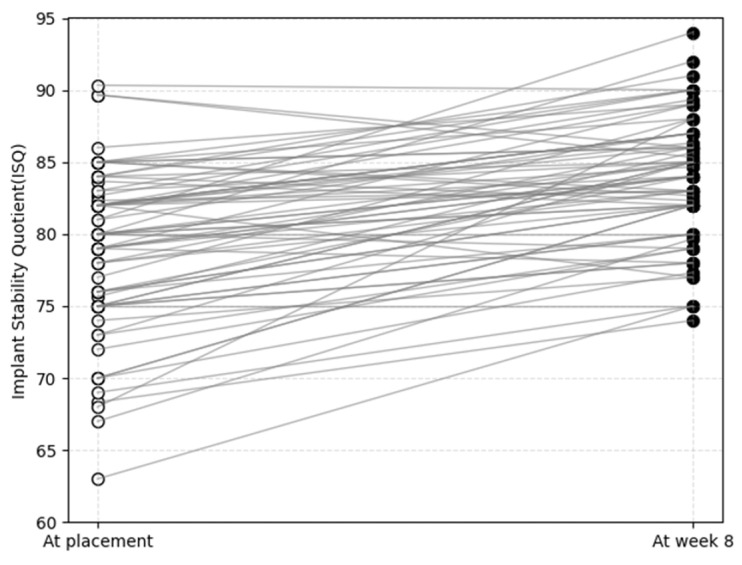
Individual implant stability quotient (ISQ) values of plasma-treated implants at baseline (placement) and after 8 weeks of healing. Each line represents the transition of a single implant from its initial ISQ (ISQ_i_) to the final ISQ at week 8 (ISQ_8_).

To compare overall trends in implant stability, initial ISQ values at placement (ISQ_i_) and final ISQ values at week 8 (ISQ_8_) were individually plotted (Figure 2). Although ISQ_i_ values showed considerable variation, ranging from 63.0 to 90.3, most converged toward higher stability by week 8, except for three cases with exceptionally high initial values (>85.0). Ultimately, all implants reached ISQ values above 74.0 at week 8. A representative case with a relatively low ISQ_i_ of 68.0 demonstrated the steepest slope of stability gain, achieving an ISQ_8_ of 88.0.

**Figure 3:**
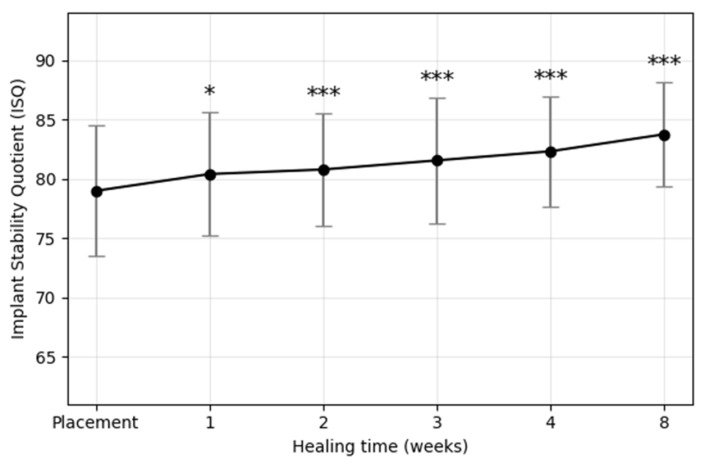
Mean implant stability quotient (ISQ) values of plasma-treated implants during the 8-week healing period. Data are presented as mean ± standard deviation. A significant time-dependent increase in ISQ values was observed, with the greatest gain occurring during the first three weeks. Asterisks indicate statistically significant differences compared with baseline (* *p* < 0.05, ** *p* < 0.01, *** *p* < 0.001).

The mean ISQ values at each postoperative time point are summarized in Figure 3 and Table 2. Overall, ISQ values showed a consistent upward trajectory throughout the 8-week healing period, without evidence of a transient stability dip. The most rapid increase occurred during the first three weeks, followed by a moderate but steady rise thereafter. Repeated-measures ANOVA confirmed a significant effect of healing time on ISQ values (*p* < 0.001), and post hoc tests showed that ISQ values at weeks 1, 2, 3, 4, and 8 were all significantly higher than baseline. On average, ISQ increased by approximately 4.8 from placement to week 8. In addition, the standard deviation gradually narrowed (±5.52 at baseline to ±4.36 at week 8), suggesting reduced variability and improved predictability of implant stability over time.

### 3.2. Initial ISQ (ISQ_i_) Ranges

For comparative analysis, the dataset was stratified into three cohorts according to primary ISQ values: (1) moderate initial stability (ISQ_i_ 65–74), (2) moderately high initial stability (ISQ_i_ 75–84), and (3) high initial stability (ISQ_i_ ≥ 85). ISQ values at each follow-up visit were labeled according to the corresponding postoperative week (ISQ_i_ to ISQ_8_). In the moderate stability group (ISQ_i_ 65–74), ISQ values increased continuously throughout the healing period, with only a transient plateau observed at week 3. Although no statistically significant differences were detected during the first two weeks, a significant increase emerged at week 3 (*p* < 0.05) and persisted through week 8. In the moderately high stability group (ISQ_i_ 75–84), ISQ values also followed a steadily increasing trajectory, with significant improvements evident from week 1 onward and maintained across all subsequent time points. Importantly, neither group exhibited a stability dip at any stage of the healing period. In contrast, implants in the high stability group (ISQ_i_ ≥ 85) demonstrated no significant change, with mean values remaining above 86 and a slight regression toward baseline by week 8.

**Figure 4:**
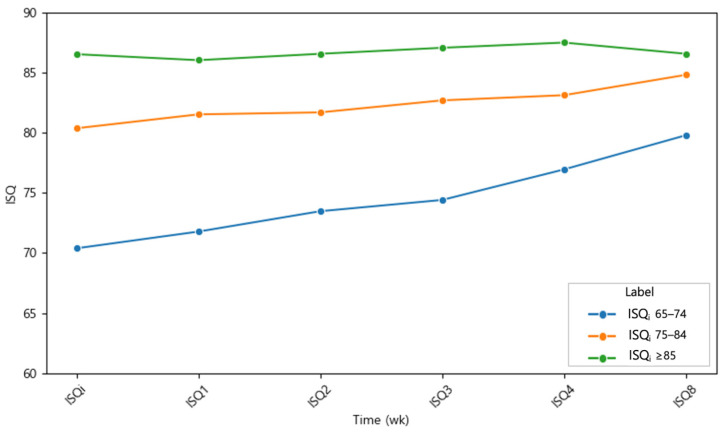
Temporal development of implant stability quotient (ISQ) values according to the initial stability groups: ISQ_i_ 65–74 (blue), ISQ_i_ 75–84 (green), and ISQ_i_ ≥ 85 (orange). Values represent mean ISQ at each time point. Standard deviations are provided in Table 4.

**Figure 5:**
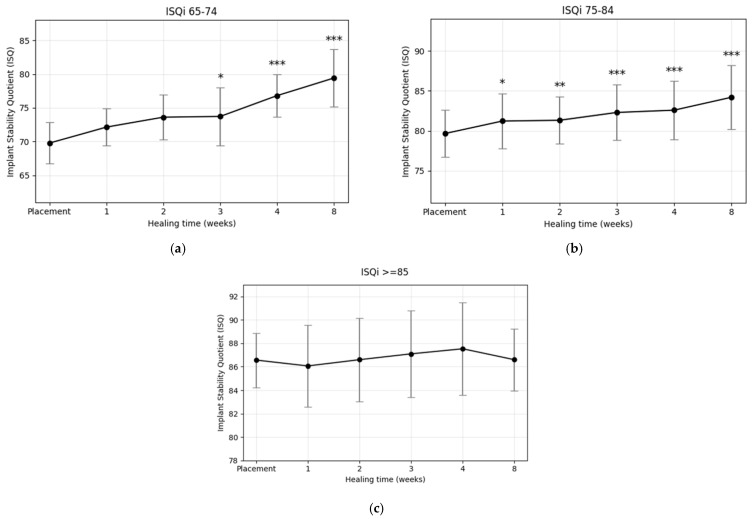
Changes in implant stability quotient (ISQ) over time by primary stability at placement. (**a**) ISQ_i_ 65–74: steepest increase, significant from week 3 onward (* *p* < 0.05, ** *p* < 0.01, *** *p* < 0.001); (**b**) ISQ_i_ 75–84: gradual but significant increase from week 1 onward; (**c**) ISQ_i_ ≥ 85: no statistically significant change, values remained consistently high.

Changes in implant stability from placement to week 8 for each ISQ_i_ group are summarized in Table 3, with mean weekly values presented in Table 4 (Figure 4 and Figure 5a–c). Paired *t*-tests confirmed that implants with ISQ_i_ ≤ 84 achieved significant improvements, whereas those with ISQ_i_ ≥ 85 did not. Specifically, the ISQ_i_ 65–74 group exhibited the greatest gain (ΔISQ = 9.64, *p* < 0.001), while the ISQ_i_ 75–84 group showed a smaller but still significant increase (ΔISQ = 4.55, *p* < 0.001).

To further characterize the dynamics of implant stability, the osseointegration speed index (OSI), defined as the monthly increase in ISQ, was calculated (Table 3). Only cohorts that demonstrated a statistically significant change between baseline (ISQ_i_) and week 8 (ISQ_8_) were included; thus, the high-stability group (ISQ_i_ ≥ 85) was excluded. OSI was calculated as (ISQ_8_ – ISQ_i_)/2, representing the mean increase in ISQ units per month over the 8-week observation period. As OSI is directly proportional to overall ISQ change, intergroup differences in OSI reflected the corresponding statistical significance observed for ISQ changes. The ISQ_i_ 65–74 group had an OSI of 6.43 ± 3.10, more than twice that of the ISQ_i_ 75–84 group (3.03 ± 2.48). This indicates that implants with lower initial stability exhibited a substantially faster rate of stability gain compared with those with moderately high initial stability.

### 3.3. Implant Location

**Figure 6:**
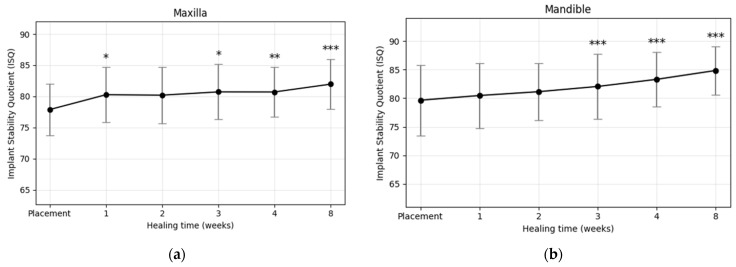
Weekly changes in implant stability quotient (ISQ) of plasma-treated implants placed in (**a**) the maxilla and (**b**) the mandible. Both groups showed significant increases during healing, with greater stability gains observed in the mandible (* *p* < 0.05, ** *p* < 0.01, *** *p* < 0.001).

A statistically significant increase in ISQ values during the healing period was observed in implants placed in both the maxilla and mandible (Figure 6a,b). Paired *t*-test results (Table 5) confirmed significant mean ISQ gains in the maxilla (ΔISQ = 4.08 ± 3.96, *p* < 0.001) and mandible (ΔISQ = 5.19 ± 4.97, *p* < 0.001). At baseline, initial ISQ values were slightly higher in the mandible (79.66 ± 6.18) compared with the maxilla (77.88 ± 4.13). This difference persisted at week 8, with mean ISQ values of 84.85 ± 4.23 in the mandible and 81.96 ± 4.04 in the maxilla. The osseointegration speed index (OSI) was also greater in the mandible (0.65 ± 0.62) than in the maxilla (0.51 ± 0.49), indicating a faster rate of stability gain over the 2-month period. Collectively, implants placed in the mandible exhibited higher primary stability and greater subsequent increases in stability than those in the maxilla (*p* < 0.001).

### 3.4. Length and Width of Implant Fixtures

The influence of implant fixture length and diameter on ISQ changes from placement to 8 weeks postoperatively was evaluated (Table 6 and Table 7). As shown in Table 6, implants with a fixture length ≤ 10 mm demonstrated a greater increase in ISQ values (ΔISQ = 5.33 ± 4.77) compared with those 11–13 mm in length (ΔISQ = 4.08 ± 4.37). The OSI was likewise higher in the shorter-length group (0.67 ± 0.60 vs. 0.51 ± 0.54), and the difference in mean ISQ at week 8 reached statistical significance (*p* < 0.05), suggesting that shorter implants may exhibit enhanced osseointegration dynamics during early healing.

Table 7 summarizes the results stratified by implant fixture diameter. Although wider-diameter implants tended to show greater increases in ISQ and higher OSI values—with 6.0 mm implants demonstrating the largest mean ISQ gain (ΔISQ = 9.00 ± 3.61) and highest OSI (1.13 ± 0.45)—these differences did not reach statistical significance. Moreover, this subgroup included only three implants (*n* = 3), and therefore the results should be interpreted with caution.

### 3.5. Final Insertion Torque Value

**Figure 7:**
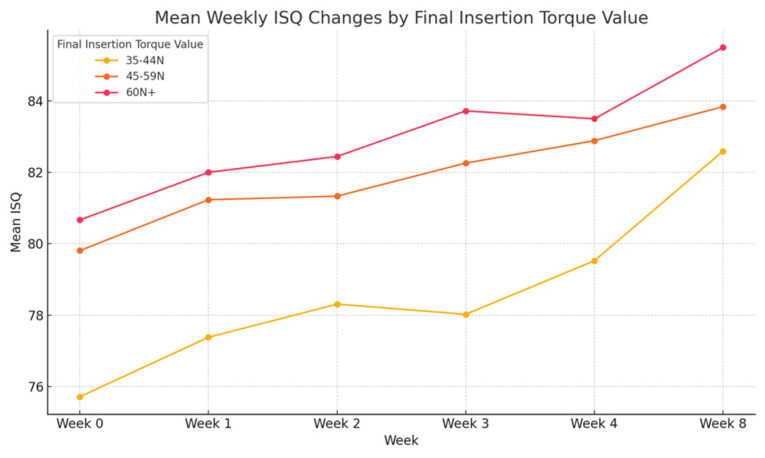
Mean weekly implant stability quotient (ISQ) changes stratified by final insertion torque. Higher torque values showed greater and more consistent stability gains.

The correlation between final insertion torque and ISQ change was evaluated (Figure 7, Table 8). Implants were stratified into three groups: 35–44 N·cm, 45–59 N·cm, and ≥60 N·cm. Across all time points, higher torque values were associated with greater ISQ measurements. The ≥60 N·cm group consistently showed the highest stability, with mean ISQ increasing from 80.67 at placement to 85.50 at week 8. By contrast, the 35–44 N·cm group began with the lowest ISQ (75.71) and reached 82.60 by week 8. Although all groups demonstrated progressive stability gains, implants placed with higher torque exhibited faster and more predictable increases in ISQ, indicating a positive correlation between primary mechanical stability and early osseointegration. Statistically significant differences among groups were observed at weeks 0, 1, 3, and 4 (*p* < 0.05). However, interpretation of the ≥60 N·cm subgroup should be made cautiously due to its limited sample size (*n* = 7).

## 4. Discussion

This retrospective single-arm cohort study quantitatively investigated the clinical efficacy of gas plasma treatment on dental implants by assessing changes in implant stability and the rate of osseointegration using the implant stability quotient (ISQ). Plasma activation aims to restore or enhance the biological activity of implant surfaces that may undergo biological aging during storage. The objective of this study was to validate, in a clinical setting, the beneficial effects of plasma-treated implants that have been consistently demonstrated in preclinical studies. As shown previously, animal experiments confirmed that plasma treatment accelerates peri-implant bone formation by introducing superhydrophilicity and removing hydrocarbon contaminants from titanium surfaces.

Plasma activation has also been proposed as a practical alternative to ultraviolet (UV)-based surface functionalization. While UV treatment can improve surface bioactivity, it is limited by higher costs, the need for crystalline packaging to permit UV penetration, and a prolonged activation process of at least three hours, making it unsuitable for chairside use [4]. By contrast, plasma treatment delivers stronger surface energy, achieves more effective removal of organic contaminants, and can be applied immediately before implant placement—an important consideration given the rapid re-adsorption of hydrocarbons from ambient air [34].

Implant stability is widely recognized as a reflective parameter of osseointegration [35]. Assessing stability across multiple time points provides clinically relevant insights into the optimal healing period for individual patients. Although several methods exist for evaluating implant stability—including push-out and pull-out tests, removal torque analysis, percussion tests, and histological examination—resonance frequency analysis (RFA) is considered the most practical and non-invasive technique for routine clinical application [36]. ISQ values ≥ 70 is generally accepted as indicative of sufficient stability for functional loading. However, because single ISQ values correlate poorly with bone quality, recent studies have emphasized the clinical importance of tracking ISQ changes over time rather than relying solely on isolated values [37]. Consistent with this approach, the present study focused on longitudinal changes in ISQ, highlighting stability progression during the healing phase rather than point measurements alone.

A critical issue during healing phase is the potential occurrence of a “stability dip,” defined as a temporary decline in implant stability caused by the gradual loss of mechanical (primary) stability before biological (secondary) stability is fully established [38]. Implants are particularly vulnerable to osseointegration failure during this period [28], and therefore, conventional protocols advise against functional loading until the dip has resolved, limiting the feasibility of immediate or early loading [26]. As stability dips are typically observed between the 2nd and 8th postoperative weeks, the present study was designed with an 8-week follow-up to detect any potential decline [39].

Surface properties are thought to play a decisive role in the occurrence and magnitude of stability dips [40]. Surface functionalization strategies such as plasma treatment enhance bone–implant integration and promote earlier biological stability, thereby compensating for the loss of mechanical stability [11]. Indeed, prior reports have shown that surface-treated implants often do not exhibit a distinct stability dip [11,39], and one study confirmed that plasma treatment specifically reduces the likelihood of a dip and accelerates stability recovery if it occurs [41]. Consistent with these reports, our study demonstrated a steady increase in ISQ values during early healing, suggesting that plasma activation may help preserve stability throughout this critical phase.

This study demonstrated a consistent time-dependent increase in implant stability following plasma surface treatment, with progressive ISQ gains throughout the 8-week healing period. This suggests that plasma activation may enhance the early healing environment and support early functional loading. Since early functional loading can instead act as a risk factor for impaired stability, the fact that implant stability was maintained and even improved further supports the beneficial effect of plasma activation. Notably, implants with relatively low initial stability (ISQ_i_ 65–74) exhibited the greatest ISQ gains and the highest OSI values, indicating that plasma treatment may be particularly beneficial in cases with suboptimal baseline conditions. All implants in this study had insertion torque values above 35 N·cm and were placed using an underdrilling protocol, both of which contributed to generally high primary stability. This uniformity limits assessment of plasma activation in truly low-torque conditions; nevertheless, the marked ISQ gains in the lower-stability subgroup suggest that plasma treatment may confer benefits beyond the mechanical stability obtained through surgical technique. Conversely, implants with very high primary stability (ISQ_i_ ≥ 85) showed minimal change, implying a ceiling effect in the context of already optimal bone–implant contact.

Site and implant-related factors also influenced outcomes. Implants placed in the mandible demonstrated significantly higher stability and faster integration compared to those in the maxilla, reflecting known differences in bone quality between the jaws. This difference is consistent with the well-established observation that mandibular bone is denser than maxillary bone, which likely contributed to the higher stability observed in mandibular sites. Although bone quality also varies between anterior and posterior regions, the present study analyzed outcomes by maxilla and mandible to ensure adequate subgroup sizes. Shorter implants (≤10 mm) also showed greater improvement in ISQ, potentially due to greater sensitivity to surface modification. Although higher insertion torque was associated with improved stability outcomes—particularly in the ≥60 N·cm group—further research with larger sample sizes is warranted to validate these findings. Overall, these results support the clinical utility of plasma surface treatment in enhancing early implant stability, with the greatest benefits likely to occur in challenging clinical scenarios.

Numerous studies have shown that primary implant stability strongly influences the development of overall stability during the healing phase [39,42,43]. Implants with moderate baseline stability generally demonstrate progressive improvement, whereas those with very high initial stability may show minimal gains or even slight reductions over time [42]. Specifically, implants with ISQ values below 60 typically exhibit substantial increases, while those with higher initial ISQ values (≥60) tend to demonstrate only minor changes or occasional declines [44,45]. In other words, implants placed in low-density bone often “catch up” in stability with those placed in denser bone during healing [43]. Consistent with these reports, the present study confirmed that implants with the lowest primary ISQ (65–74) exhibited the greatest increase in stability over the 8-week observation period.

High initial stability is generally associated with elevated insertion torque, which, when excessive, may induce compression necrosis [46]. This phenomenon arises from excessive mechanical stress at the bone–implant interface, compromising blood flow and potentially impairing early-phase healing [47]. Furthermore, underpreparation of the osteotomy site can exacerbate this effect by causing irreversible microdamage to surrounding bone [48]. These mechanisms may help explain the relatively limited ISQ gains observed in the high initial stability group in the present study.

To compensate for the absence of a control group in this single-arm study, relevant literature on untreated implants without post-packaging surface modification was reviewed for comparison. In particular, Suzuki et al. conducted a prospective human cohort study in which 33 photofunctionalized implants were immediately loaded in the maxilla and monitored for up to 3 months. They observed consistently rising ISQ values, elimination of the stability dip, and markedly higher OSI values (6.3 for ISQ_i_ 65–70; 3.1 for ISQ_i_ 71–75) compared with conventional untreated implants reported in the literature, which generally showed OSI values below 1.0 [26]. In the present study, plasma-activated implants similarly exhibited substantially higher OSI values (6.43 for ISQ_i_ 65–74; 3.03 for ISQ_i_ 75–84) and final ISQ values at 8 weeks (79.42–86.60), despite the shorter observation period [26]. These parallels reinforce the interpretation that surface activation—whether by UV photofunctionalization or cold plasma—can accelerate and reinforce osseointegration, particularly in implants with lower initial stability.

Nevertheless, this study has several limitations. First, as a retrospective analysis, both implant- and surgery-related factors could not be standardized. This heterogeneity limits the generalizability of our findings, although it also reflects real-world clinical practice. Well-designed comparative studies with standardized implant systems and surgical protocols will be necessary to more precisely evaluate the true impact of plasma treatment on implant stability. Second, bone quality was not consistently or objectively assessed. Given the subjectivity of radiographic bone quality assessments, we emphasized ISQ outcomes, yet the lack of standardized bone quality data remains a limitation. Third, the lack of a control group limits causal inference; the present findings should therefore be interpreted as preliminary and require validation in prospective controlled trials. Finally, long-term clinical outcomes such as survival and peri-implant tissue response were not systematically collected, restricting interpretation to short-term ISQ data. Therefore, our findings should be regarded as supportive preliminary evidence rather than a substitute for such clinical endpoints, and further prospective trials with standardized protocols are required to validate the long-term clinical utility of plasma treatment.

## 5. Conclusions

This retrospective cohort study demonstrated that chairside plasma surface treatment was associated with progressive improvements in early implant stability, as reflected by steady ISQ gains throughout the 8-week healing period. Plasma activation may enhance the biological performance of dental implant surfaces by removing hydrocarbon contaminants and restoring superhydrophilicity, thereby facilitating protein adsorption, cellular attachment, and early osteogenic activity. Notably, implants with lower initial stability (ISQ_i_ 65–74) exhibited the greatest ISQ gains and the highest OSI values, while those with very high initial stability (ISQ_i_ ≥ 85) showed minimal change. Stability improvements were also greater in mandibular implants, shorter fixtures, and those with higher insertion torque. These results suggest that plasma activation may support early stability under clinically challenging conditions; however, the findings are limited to short-term ISQ outcomes and do not extend to long-term clinical endpoints. Prospective randomized controlled trials with standardized protocols and longer follow-up are required to expand upon these results.

## Figures and Tables

**Table 1:** Patient and implant data. The distribution of torque value at initial placement and implantation site is shown.

	Patients			Implants			Initial Torque (N·cm)		
Number	Age Range	Sex		Total Number	Maxilla	Mandible	35 N–40 N	45 N–50 N	>50 N
		Female	Male						
47	38–86	20 (43%)	27 (57%)	73	28 (38%)	45 (62%)	14	52	7

**Table 2:** Mean ISQs ± SDs during the healing period.

	Time (Week)					
	Placement	1	2	3	4	8
ISQ	78.97 ± 5.52	80.39 ± 5.23	80.78 ± 4.78	81.54 ± 5.28	82.31 ± 4.64	83.74 ± 4.36
95% CI	77.70–80.24	79.19–81.59	79.68–81.88	80.33–82.75	81.25–83.37	82.74–84.74

**Table 3:** Results of *t*-test analysis comparing ISQ values at baseline and at week 8 across three initial ISQ categories. Values are presented as mean ± SD. 95% confidence intervals (CI) for ΔISQ and OSI were as follows: (1) ISQ_i_ 65–74: ΔISQ 6.60–12.68, OSI 4.40–8.46; (2) ISQ_i_ 75–84: ΔISQ 3.56–5.54, OSI 2.37–3.69; (3) ISQ_i_ ≥ 85: ΔISQ −1.89–1.95. Statistical significance was assessed with repeated-measures ANOVA and Bonferroni correction (*** *p* < 0.001).

Initial Stability Range	Number of Implants	At Placement	At Week 8	Change (ΔISQ)	OSI
**ISQ_i_ 65–74**	9	69.78 ± 3.06	79.42 ± 4.23	9.64 ± 4.65 ***	6.43 ± 3.10
**ISQ_i_ 75–84**	54	79.65 ± 2.93	84.20 ± 3.98	4.55 ± 3.73 ***	3.03 ± 2.48
**ISQ_i_ ≥ 85**	10	86.57 ± 2.32	86.60 ± 2.62	0.03 ± 3.10	NA

**Table 4:** Mean ISQs ± SDs in each group over time.

	Time (Week)					
Groups	Placement	1	2	3	4	8
**ISQ_i_ 65–74**	69.77 ± 3.06	72.14 ± 2.74	73.61 ± 3.34	73.72 ± 4.29	76.81 ± 3.14	79.42 ± 4.23
**ISQ_i_ 75–84**	79.65 ± 2.93	81.22 ± 3.46	81.32 ± 2.97	82.29 ± 3.49	82.58 ± 3.64	84.20 ± 3.98
**ISQ_i_ ≥ 85**	86.57 ± 2.32	86.07 ± 3.50	86.60 ± 3.55	87.10 ± 3.71	87.53 ± 3.95	86.60 ± 2.62

**Table 5:** Results of *t*-test analysis comparing ISQ values at baseline and at week 8 in the maxilla and mandible. Values are presented as mean ± SD. Paired *t*-tests were used for within-group comparisons. *** *p* < 0.001.

Jaw Location	Number of Implants	At Placement	At Week 8	Change (ΔISQ)	OSI
**Maxilla**	28	77.88 ± 4.13	81.96 ± 4.04	4.08 ± 3.96 ***	0.51 ± 0.49
**Mandible**	45	79.66 ± 6.18	84.85 ± 4.23	5.19 ± 4.97 ***	0.65 ± 0.62

**Table 6:** Results of *t*-test analysis of changes in ISQ values according to implant length. Asterisks indicate statistically significant differences compared with baseline (* *p* < 0.05, ** *p* < 0.01, *** *p* < 0.001).

Length	Number of Implants	At Placement	At Week 8	Change (ΔISQ)	OSI
**≤10 mm**	40	79.72 ± 4.76	85.05 ± 4.31 *	5.33 ± 4.77	0.67 ± 0.60
**11 mm ≤ 13 mm**	33	78.08 ± 6.29	82.16 ± 3.93 *	4.08 ± 4.37	0.51 ± 0.54

**Table 7:** Results of *t*-test analysis of changes in ISQ values according to implant fixture diameter.

Diameter	Number of Implants	At Placement	At Week 8	Change (ΔISQ)	OSI
**4.0**	11	78.15 ± 5.36	81.36 ± 3.75	3.21 ± 4.96	0.40 ± 0.62
**4.5**	21	79.84 ± 4.55	83.43 ± 3.56	3.59 ± 3.12	0.45 ± 0.39
**5.0**	38	78.72 ± 6.25	84.25 ± 4.70	5.54 ± 5.01	0.69 ± 0.63
**6.0**	3	79.22 ± 3.24	88.22 ± 3.34	9.00 ± 3.61	1.13 ± 0.45

**Table 8:** Comparison of ISQ value changes by final insertion torque value using *t*-test analysis. Values are presented as mean ± SD. One-way ANOVA with Bonferroni post hoc tests was applied for group comparisons. * *p* < 0.05; ** *p* < 0.01; *** *p* < 0.001.

Final Insertion Torque Value	Number of Implants	At Placement	At Week 8	Change (ΔISQ)
**35–44 N·cm**	14	75.14 ± 5.53	82.60 ± 3.95 *	5.33 ± 4.77 *
**45–59 N·cm**	52	79.66 ± 5.44	83.87 ± 4.56 *	4.08 ± 4.37 *
**≥60 N·cm**	7	80.43 ± 4.08	85.14 ± 3.53 *	4.71 ± 3.94 *

## Data Availability

The original contributions presented in this study are included in the article. Further inquiries can be directed to the corresponding author.

## References

[1] Yeo I.S. (2020). Modifications of dental implant surfaces at the micro- and nano-level for enhanced osseointegration. Materials.

[2] Buser D., Schenk R.K., Steinemann S., Fiorellini J.P., Fox C.H., Stich H. (1991). Influence of surface characteristics on bone integration of titanium implants. J. Biomed. Mater. Res..

[3] Jang T.S., Jung H.D., Kim S., Moon B.S., Beak J., Park C., Song J., Kim H.E. (2017). Multiscale porous titanium surfaces via a two-step etching process for improved mechanical and biological performance. Biomed. Mater..

[4] Lee H., Jeon H.J., Jung A., Kim J., Kim J.Y., Lee S.H., Kim H., Yeom M.S., Choe W., Gweon B. (2022). Improvement of osseointegration efficacy of titanium implant through plasma surface treatment. Biomed. Eng. Lett..

[5] Lee J.H., Ogawa T. (2012). The biological aging of titanium implants. Implant Dent..

[6] Minamikawa H., Att W., Ikeda T., Hirota M., Ogawa T. (2016). Long-term progressive degradation of the biological capability of titanium. Materials.

[7] Sousa S.R., Lamghari M., Sampaio P., Moradas Ferreira P., Barbosa M.A. (2008). Osteoblast adhesion and morphology on TiO_2_ depends on the competitive preadsorption of albumin and fibronectin. J. Biomed. Mater. Res. A.

[8] Att W., Hori N., Takeuchi M., Ouyang J., Yang Y., Anpo M., Ogawa T. (2009). Time dependent degradation of titanium osteoconductivity: An implication of biological aging of implant materials. Biomaterials.

[9] Yoshinari M., Matsuzaka K., Inoue T., Oda Y., Shimono M. (2002). Bio-functionalization of titanium surfaces for dental implants. Mater. Trans..

[10] Tsujita H., Nishizaki H., Miyake A., Takao S., Komasa S. (2021). Effect of plasma treatment on titanium surface on the tissue surrounding implant material. Int. J. Mol. Sci..

[11] Strnad J., Urban K., Povysil C., Strnad Z. (2008). Secondary stability assessment of titanium implants with an alkali-etched surface: A resonance frequency analysis study in beagle dogs. Int. J. Oral Maxillofac. Implant..

[12] Coelho P.G., Giro G., Teixeira H.S., Marin C., Witek L., Thompson V.P., Tovar N., Silva N.R.F.A. (2012). Argon based atmospheric pressure plasma enhances early bone response to rough titanium surfaces. J. Biomed. Mater. Res. A.

[13] Wu C., Yang M., Ma K., Zhang Q., Bai N., Liu Y. (2023). Improvement of implant osseointegration through nonthermal Ar/O_2_ plasma. Dent. Mater. J..

[14] Pedrosa P., Chappé J.M., Fonseca C., Vaz F. (2010). Plasma surface modification of polycarbonate and poly(propylene) substrates for biomedical electrodes. Plasma Process. Polym..

[15] Kim J.Y., Lee J.H., Park J.K., Choi Y.C. (2008). Effect of N_2_, Ar, and O_2_ plasma treatments on surface properties of metals. J. Appl. Phys..

[16] Liu S., Xu L., Zhang T., Ren G., Yang Z. (2010). Oxidative stress and apoptosis induced by nanosized titanium dioxide in PC12 cells. Toxicology.

[17] Ujino D., Nishizaki H., Higuchi S., Komasa S., Okazaki J. (2019). Effect of plasma treatment of titanium surface on biocompatibility. Appl. Sci..

[18] Kwon J.S., Cho W.T., Lee J.H., Joo J.Y., Lee J.Y., Lim Y., Jeon H.J., Huh J.B. (2024). Prospective randomized controlled clinical trial to evaluate the safety and efficacy of ACTILINK plasma treatment for promoting osseointegration and bone regeneration in dental implants. Bioengineering.

[19] Lee J.W., Park J.H., Kim Y.H. (2020). Effect of atmospheric pressure plasma treatment on the titanium surface and tissue compatibility. Appl. Sci..

[20] Minati L., Migliaresi C., Lunelli L., Viero G., Dalla Serra M., Speranza G. (2017). Plasma assisted surface treatments of biomaterials. Biophys. Chem..

[21] Roach P., Farrar D., Perry C.C. (2005). Interpretation of protein adsorption: Surface-induced conformational changes. J. Am. Chem. Soc..

[22] Masaki C., Schneider G.B., Zaharias R., Seabold D., Stanford C. (2005). Effects of implant surface microtopography on osteoblast gene expression. Clin. Oral Implants Res..

[23] Alqutaibi A.Y., Aljohani A., Alduri A., Masoudi A., Alsaedi A.M., Al-Sharani H.M., Farghal A.E., Alnazzawi A.A., Aboalrejal A.N., Mohamed A.-A.H. (2023). The effectiveness of cold atmospheric plasma (CAP) on bacterial reduction in dental implants: A systematic review. Biomolecules.

[24] Kahm S.H., Lee S.H., Lim Y., Jeon H.J., Yun K.I. (2024). Osseointegration of dental implants after vacuum plasma surface treatment in vivo. J. Funct. Biomater..

[25] Canullo L., Tallarico M., Peñarrocha-Oltra D., Monje A., Wang H.L., Peñarrocha-Diago M. (2016). Implant abutment cleaning by plasma of argon: Five-year follow-up of a randomized controlled trial. J. Periodontol..

[26] Suzuki S., Kobayashi H., Ogawa T. (2013). Implant stability change and osseointegration speed of immediately loaded photofunctionalized implants. Implant Dent..

[27] Albrektsson T., Wennerberg A. (2019). On osseointegration in relation to implant surfaces. Clin. Implant Dent. Relat. Res..

[28] Raghavendra S., Wood M.C., Taylor T.D. (2005). Early wound healing around endosseous implants: A review of the literature. Int. J. Oral Maxillofac. Implants.

[29] Plasmapp Inc. (2024). ACTILINK Product Specification Sheet. https://www.plasmapp.com.

[30] Nevins M., Chen C.Y., Parma Benfenati S., Kim D.M. (2023). Gas plasma treatment improves titanium dental implant osseointegration: A preclinical in vivo experimental study. Bioengineering.

[31] Makary C., Menhall A., Lahoud P., An H.-W., Park K.-B., Traini T. (2023). Nanostructured calcium-incorporated surface compared to machined and SLA dental implants: A split-mouth randomized case/double-control histological human study. Nanomaterials.

[32] Kim J.C., Lee M., Yeo I.-S.L. (2022). Three interfaces of the dental implant system and their clinical effects on hard and soft tissues. Mater. Horiz..

[33] Megagen Implant Co., Ltd. (2021). MEGA ISQ™ User Manual.

[34] Berger M.B., Bosh K.B., Cohen D.J., Boyan B.D., Schwartz Z. (2021). Benchtop plasma treatment of titanium surfaces enhances cell response. Dent. Mater..

[35] Brunski J.B. (1992). Biomechanical factors affecting the bone–dental implant interface. Clin. Mater..

[36] Atsumi M., Park S.H., Wang H.L. (2007). Methods used to assess implant stability: Current status. Int. J. Oral Maxillofac. Implants.

[37] Fu M.W., Fu E., Lin F.G., Chang W.J., Hsieh Y.D., Shen E.C. (2017). Correlation between resonance frequency analysis and bone quality assessments at dental implant recipient sites. Int. J. Oral Maxillofac. Implants.

[38] Sennerby L., Meredith N. (2008). Implant stability measurements using resonance frequency analysis: Biological and biomechanical aspects and clinical implications. Periodontology 2000.

[39] Balshi S.F., Allen F.D., Wolfinger G.J., Balshi T.J. (2005). A resonance frequency analysis assessment of maxillary and mandibular immediately loaded implants. Int. J. Oral Maxillofac. Implants.

[40] Simůnek A., Kopečka D., Brazda T., Strnad I., Čapek L., Slezák R. (2012). Development of implant stability during early healing of immediately loaded implants. Int. J. Oral Maxillofac. Implants.

[41] Stacchi C., Rapani A., Montanari M., Martini R., Lombardi T. (2025). Effect of Vacuum Plasma Activation on Early Implant Stability: A Single-Blind Split-Mouth Randomized Clinical Trial. J. Oral Maxillofac. Res..

[42] Simunek A., Strnad J., Kopecka D., Brazda T., Pilathadka S., Chauhan R., Slezak R., Capek L. (2010). Changes in stability after healing of immediately loaded dental implants. Int. J. Oral Maxillofac. Implants.

[43] Friberg B., Sennerby L., Meredith N., Lekholm U. (1999). A comparison between cutting torque and resonance frequency measurements of maxillary implants: A 20-month clinical study. Int. J. Oral Maxillofac. Surg..

[44] Glauser R., Sennerby L., Meredith N., Lundgren A., Gottlow J., Hämmerle C.H.F. (2004). Resonance frequency analysis of implants subjected to immediate or early functional occlusal loading: Successful vs. failing implants. Clin. Oral Implants Res..

[45] Makary C., Rebaudi A., Sammartino G., Naaman N. (2012). Implant primary stability determined by resonance frequency analysis: Correlation with insertion torque, histologic bone volume, and torsional stability at 6 weeks. Implant Dent..

[46] Do Vale Souza J.P., de Moraes Melo Neto C.L., Piacenza L.T., Da Silva E.V., de Melo Moreno A.L., Penitente P.A., Brunetto J.L., Dos Santos D.M., Goiato M.C. (2021). Relation between insertion torque and implant stability quotient: A clinical study. Eur. J. Dent..

[47] Ramesh R., Sasi A., Mohamed S.C., Joseph S.P. (2024). “Compression Necrosis”—A cause of concern for early implant failure? Case report and review of literature. Clin. Cosmet. Investig. Dent..

[48] Stocchero M., Toia M., Cecchinato D., Becktor J.P., Coelho P.G., Biomechinical J.R. (2016). Biologic, and clinical outcomes of undersized implant surgical preparation: A systematic review. Int. J. Oral Maxillofac. Implants.

